# Quality-of-life among women with breast cancer: application of the international classification of functioning, disability and health model

**DOI:** 10.3389/fpsyg.2024.1318584

**Published:** 2024-02-01

**Authors:** Alham Al-Sharman, Areen Al-Sarhan, Ala Aburub, Raid Shorman, Ali Bani-Ahmad, Catherine Siengsukon, Wegdan Bani Issa, Dana N. Abdelrahim, Heba Hijazi, Hanan Khalil

**Affiliations:** ^1^Department of Physiotherapy, College of Health Sciences, University of Sharjah, Sharjah, United Arab Emirates; ^2^Department of Rehabilitation Sciences, Faculty of Applied Medical Sciences, Jordan University of Science and Technology, Irbid, Jordan; ^3^Research Institute of Medical and Health Sciences, University of Sharjah, Sharjah, United Arab Emirates; ^4^Department of Allied Medical Sciences, Faculty of Applied Medical Sciences, Jordan University of Science and Technology, Irbid, Jordan; ^5^Department of Physical Therapy, Rehabilitation Science, & Athletic Training, University of Kansas Medical Center, Kansas City, KS, United States; ^6^Department of Nursing, College of Health Sciences, University of Sharjah, Sharjah, United Arab Emirates; ^7^Department of Health Care Management, College of Health Sciences, University of Sharjah, Sharjah, United Arab Emirates; ^8^Department of Health Management and Policy, Faculty of Medicine, Jordan University of Science and Technology, Irbid, Jordan; ^9^Department of Rehabilitation Sciences, College of Health Sciences, QU Health, Doha, Qatar

**Keywords:** breast cancer, quality of life, predictor factors, ICF model, Jordan

## Abstract

**Background:**

This study aimed to identify the factors that influence Breast Cancer (BC) women’s quality of life (QoL) based on the International Classification of Functioning, Disability and Health (ICF) framework.

**Method:**

A cross-sectional study was conducted among 188 women with BC. The dependent variable, QoL, was measured using the Quality of Life Index (QLI-c). The independent variables were measured using the following Arabic-validated questionnaires: Pittsburgh Sleep Quality Index (PSQI), Female Sexual Function Index (FSFI), Modified Fatigue Impact Scale (MFIS), Hospital Anxiety and Depression Scale (HADS), and the International Physical Activity Questionnaire (IPAQ).

**Results:**

There was a significant positive correlation between monthly income (*r* = 0.17, *p* = 0.016) and QoL, and significant negative correlation between the stage of disease (*r* = −0.221, *p* = 0.002) and duration of first diagnosis (*r* = −0.280, *p* = 0.004) with QoL. Poor sleep quality, sexual dysfunction, fatigue, depression, and anxiety had significant negative correlations with QoL (*p* < 0.01). Multiple regression analysis revealed that among the various factors that might affect QoL, sexual dysfunction, poor sleep quality, depression, and anxiety were significant predictors of QoL (*p* ≤ 0.05).

**Conclusion:**

The ICF provided an excellent framework to explore the factors influencing QoL among women with BC. This study has given evidence for the relationship of demographic, clinical, and body functional factors with QoL among women with BC. Interestingly, sexual dysfunction, poor sleep quality, depression, and anxiety factors are predictors of QoL. Awareness of these factors that predict QoL will guide healthcare professionals to improve the health and QoL of BC women.

## 1 Introduction

Globally, breast cancer (BC) is the most common cancer among women and the second most common type of all cancers (Bray et al., 2020). In 2018, approximately 2.1 million people worldwide were diagnosed with breast cancer, and approximately 627,000 individuals died of this disease (Bray et al., 2020). According to the World Health Organization (WHO), breast cancer is the most common cancer type in Jordan with an incidence rate of 20.8% (WHO, 2020). The most recent Jordanian National Cancer Registry reported a 978 breast cancer cases among Jordanians with a crude incidence rate of 31.8 per 100,000 (Non-Communicable Diseases Directorate Jordan Cancer Registry, 2012). Improvements in early detection and advanced technologies in treatment have led to an increased survival rate for BC patients (Merino Bonilla et al., 2017).

Women with BC experience several physical, psychological, and social consequences (Boquiren et al., 2013; Iddrisu et al., 2020). Fatigue, pain, lymphedema, sexual dysfunctions, and sleep impairments are frequently reported as physical consequences of BC (Taghian et al., 2014; Schmidt et al., 2018). In addition, psychological consequences are reported such as fear, anxiety, depression, and decreased life satisfaction (Maass et al., 2019). These consequences negatively impact health and quality of life (QoL) (Campbell-Enns and Woodgate, 2015).

Quality of life has become a prominent issue in the current healthcare system to assess the subjective perception of individuals’ life (Megari, 2013). Investigating QOL in people with chronic diseases as per BC may help improve patient health as it might guide policies, interventions, and services (Bamm et al., 2013). Furthermore, investigating QOL is very important for improving clinical research and clinical practice. However, the concept of QoL is not universally agreed upon; it has evolved over time, gaining depth across various fields (Fayers and Machin, 2013). It is best understood as multifaceted, highlighting how individuals perceive their current mental state. It encompasses environmental, social, psychological, and physical dimensions. According to the WHO, QoL is an individual’s outlook on their life within the framework of their values and culture. It involves personal aspirations, expectations, and concerns, representing an overall sense of life contentment that’s subjectively assessed based on perceived significance (Schalock, 2004). This definition incorporates aspects of a biopsychosocial model and is influenced by cognitive factors. Numerous facets of daily life contribute, including emotional responses to events, disposition, and satisfaction with both work and personal relationships.

Overall, the underlying contributing factors which could affect QoL among women with BC have been reported in many studies across different countries and many factors have been proposed (El Haidari et al., 2020). However, factors associated with QoL reported in various European and north American studies may not necessarily align with those affecting QoL among Jordanian women with BC. This disparity arises from differences in patients’ goals, expectations, concerns, as well as variations in religious beliefs, financial status, healthcare, and social systems. Interestingly, Jordanian women’s practices and perceptions are predominantly guided by their religious beliefs (Koburtay et al., 2023). Therefore, understanding the factors that affect the QoL among Jordanian women with BC would positively impact the quality of care provided for these individuals and contribute to the knowledge of the experiences of BC individuals worldwide.

While multiple factors have been associated with QoL among women with BC, the findings have been inconsistent. For example, researchers have found that QoL with BC appears to be influenced by age, marital status, medical complications, self-perceived health, sleep impairments, depression, and anxiety (Triberti et al., 2019; Mokhatri-Hesari and Montazeri, 2020). However, other researchers have not found significant relationships between these factors (Vogtmann et al., 2013; Li et al., 2020). In addition to this controversial matter, we initiated this study among Jordanian women diagnosed with breast cancer due to various motivating factors. These reasons encompass the elevated occurrence of breast cancer among Jordanian women, the scarcity of published research exploring the determinants linked to QoL in this demographic, the absence of comprehensive inclusion of potential factors (such as sexual dysfunction, sleep disturbances, and physical activity) that may impact QoL, the pursuit to enhance our comprehension of methods to ameliorate QoL within this community, and the utilization of an international framework to delineate outcomes from our ongoing investigations. Using an international framework to describe the results of our current studies.

The International Classification of Functioning, Disability, and Health (ICF) is a well-known framework that provide information about function and disability. This model also helps to understand the health status of the individuals in the context of the change in their body function, activity, participation as well as the environmental factors. The ICF describes the individual’s QoL as a changing state of functioning that may be affected by the individual’s health condition. Information that is obtained when implementing ICF in several clinical settings gives a meaningful picture of the health experience of an individual, which can be used for health management decisions (Cieza et al., 2008).

In the realm of breast cancer, the ICF model offers a comprehensive lens to comprehend the multifaceted impact of the disease. It delves beyond the medical aspects, embracing a broader spectrum of influences on an individual’s well-being. For those navigating breast cancer, the ICF model can be utilized across several domains: body functions and structures (evaluating the physical and physiological impacts of breast cancer and its treatments, such as pain, fatigue, and alterations in bodily functions); activities and Participation (assessing the limitations patients encounter in daily tasks, work, social interactions, and engagement in community life due to the effects of the disease and treatments); environmental factors (considering the influence of the physical, social, and attitudinal surroundings on a patient’s experience, including access to healthcare services, support networks, and societal perceptions of cancer). Personal factors are not classified under the ICF classification system as its not oded, however, some examples of personal factors are provided to be considered by healthcare providers.

To our knowledge, limited quantitative information on the quality of life of breast cancer patients in Jordan has been published utilizing the ICF model. So, this study aimed to expand previous research that examined factors associated with QoL among women diagnosed with BC in Jordan by using the ICF model. By employing the ICF model in breast cancer, healthcare professionals gain a comprehensive understanding of the challenges patients confront beyond the illness itself. This framework aids in tailoring interventions to address specific impairments, support daily functioning, and encourage participation while considering the unique context of each patient and the environmental factors impacting their breast cancer journey. Integrating the ICF model fosters a more nuanced approach to care, emphasizing the patient’s individual experience and promoting patient-centered strategies for support and rehabilitation.

## 2 Methods

### 2.1 Study designs and participants

This is a cross-sectional study that was conducted on a convenience sample of 188 Jordanian women with BC recruited from three different sites (the radiation oncology department at Al-Bashir Hospital, Amman, Jordan, and the chemotherapy oncology unit at King Abdullah University Hospital (KAHU) and Princess Basma Hospital, Irbid, Jordan). Researchers approached the study participants in the general waiting area of the outpatient clinics, providing details about the study. They were given a description of the study and were asked if they would be willing to participate in the study. Participants were assured that participation is voluntary and that their personal information will be anonymized to ensure confidentiality. The participants were eligible for this study if they were women living in Jordan, aged 18 or above, and diagnosed with breast cancer. We excluded women who were unable to follow the instructions. Approval for this study was obtained by the Institutional Review Board (IRB) at the Jordan University of Science and Technology (JUST), IRB at the King Abdullah University Hospital (KAUH), and the Scientific Research Ethics Committee at the Jordanian Ministry of Health.

### 2.2 Procedure and outcome measures

From December 2020 to April 2021, women with BC who visited the target hospitals were recruited in this study if they met the inclusion criteria. The research assistant conducted interviews with participants and completed the informed consent process before their enrolment in the study. All participants received detailed explanations about the study. Those who agreed to take part were invited to complete questionnaires in a serene, distraction-free environment. This setting aimed to enhance participants’ focus and encourage free and confidential responses. The investigator administered the questionnaires during face-to-face interviews. The information collected was categorized into impairment, activity, participation, and contextual factors. Impairment was assessed by the clinical characteristics of the BC (including the stage of BC, onset of the diagnosis and having surgery or not) and the presence of complications (including depression, anxiety, fatigue, sleep impairment, sexual dysfunction). Physical activity level was assessed to reflect participation. Contextual factors include age, marital status, number of family members, educational level, income level (World Salaries, 2024) and employment status (whether they are employees or not).

### 2.3 Outcome measures

#### 2.3.1 Quality of life

The Quality of Life Index-Cancer (QLI-c) was used to assess QoL. QLI-c consists of two portions. The first portion includes questions about participants’ satisfaction with various aspects of their life; the second portion assesses the importance of those aspects. The total score ranges from 0 to 30 and a higher score indicates better QoL. The Arabic version of the Quality of Life Index is highly reliable and has sufficient content validity for measuring quality of life of Arabic-speaking clients (Halabi, 2006; Omar and Alnahdi, 2023). The Cronbach’s alpha coefficient for the Arabic PSQI was 0.97, demonstrating acceptable reliability (Halabi, 2006).

#### 2.3.2 Sleep quality

The Arabic version of the Pittsburgh Sleep Quality Index (PSQI) (Al Maqbali et al., 2020) was used to assess sleep quality. PSQI is a self-reported 19-item questionnaire. The items of PSQI are combined to give seven domains (subjective sleep quality, sleep latency, sleep duration, habitual sleep efficiency, sleep disturbances, use of sleep medication, and daytime dysfunction). Scoring for each item ranges from 0 to 3, where 0 indicates no difficulty, and 3 is severe difficulty. The total score of PSQI is calculated by combining seven domain scores, ranging from 0 to 21. A higher score on PSQI indicates poor sleep quality, with a cut-off point of 5, total of 5 or less points considered as good sleep quality, while a score of 5 and above considered as poor sleep quality (Suleiman et al., 2010). The Arabic version of the PSQI demonstrated adequate reliability and validity for assessing sleep quality in Arabic-speaking patients diagnosed with cancer (Al Maqbali et al., 2020). The Cronbach’s alpha coefficient for the Arabic PSQI was 0.77, demonstrating acceptable reliability (Al Maqbali et al., 2020).

#### 2.3.3 Anxiety and depression

The Hospital Anxiety and Depression Scale (HADS) was used to evaluate the level of anxiety and depression (Spielberger, 2013; Stern, 2014). It is a 3-point Likert-type scale consisting of 14 items, 7 items for anxiety, and 7 items for depression. The total score of each subscale is out of 21. A higher score indicates a high level of anxiety and depression. A score of ≥ 11 is considered a clinically significant disorder, whereas a score between 8 and 10 suggests a mild disorder (Zigmond and Snaith, 1983). The Arabic version of HADS is reliable and valid (Terkawi et al., 2017). The scale showed a good Cronbach’s alpha equal to 0.95 among female cancer Survivors (Durosini et al., 2021).

#### 2.3.4 Sexual function

The Female Sexual Function Index (FSFI) was to measure sexual function (Rosen et al., 2000; Masjoudi et al., 2019). The FSFI covers six domains of sexual function: (sexual desire, sexual arousal, lubrication, orgasm, satisfaction, and pain). The total score of FSFI ranges from 2 to 36. A higher score indicates better sexual function. The cutoff score was ≤ 26.0 indicating female sexual dysfunctions. FSFI has excellent internal consistency reliability (0.94) in cancer survivors (Bartula and Sherman, 2015). The initial FSFI validation study reported excellent reliability for the FSFI total score (Cronbach’s alpha 0.97) and subscales (Cronbach’s alpha range, from 0.89 to 0.96), (Rosen et al., 2000).

#### 2.3.5 Fatigue

Fatigue was assessed using the Modified Fatigue Impact Scale (MFIS) (Lundgren-Nilsson et al., 2019). The MFIS is a 21-item instrument that measures the impact of fatigue over the past 4 weeks on activities of daily living. FSFI covers three domains: physical, cognitive, and psychological. Respondent rate on a 5-point Likert-type scale is the frequency of fatigue symptoms within the past 4 weeks. Item rated from (0) never to (4) always. The scoring provides a total fatigue score with a range from 0 to 84. A higher score indicates greater fatigue symptoms, the score of 38 considered as the cut-off point for scoring. The valid Arabic version of MFIS was used in this study (Alawami and Abdulla, 2021).

#### 2.3.6 Physical activity level

Activity and participation were measured by assessing the physical activity. The Short form of the International Physical Activity Questionnaire (IPAQ-short form) (Craig et al., 2003) was used which has 7 items assessing physical activity during the 7 past days by questioning the number of days and number of hours people spent walking, performing moderate or vigorous levels of physical activities. The IPAQ is widely used to assess physical activity status; it’s been validated in different populations and different countries (Burke and Sabiston, 2010; Turner et al., 2011; Burke et al., 2012). Additionally, it has high-test re-test reliability (Al Maqbali et al., 2022) and has previously shown utility in women with BC (Hsiao et al., 2019).

### 2.4 Data analysis

The statistical package for social science (SPSS) version 25 was utilized for all data entry in this study. Descriptive statistics (mean, percentage, standard deviation) were used to describe socio-demography and clinical characteristics and the score of all outcome measures. Correlations between variables were detected by the Pearson correlation coefficients to identify which factors could be included in the regression analysis. For example, if two explanatory variables have a high correlation (correlation greater than 0.7) we only include one variable in the regression analysis. Simple linear regression was first used to identify potential factors associated with QoL and only significant variables (*p*-value less than 0.05) were included in the multiple regressions. *P*-value ≤0.05 was considered statically significant for all analyses.

## 3 Results

### 3.1 Demographic and clinical characteristics results

One hundred and eighty-eight (*n* = 188) women with BC completed the study. The mean age of the sample was 52.14 ± 9.83. The level of education of half of the participants (54.1%) was intermediate and high school. More than half of the women in the sample were married (67.9%) and the mean number family members was 4.83 ± 2.23. Only 15.2% of women were workers. About half of women (54.2%) have a medium level of monthly income, while (42%) of women half a low level of monthly income. Regarding BC staging, 40.85% of women had stage III and (29.6%) of women had stage IV. More than half of the participants (62.2%) had been diagnosed >12 months. Regarding treatment, the majority of participants (83.2%) underwent breast surgery. The demographic and clinical characteristics are shown in Table 1.

**Table 1:** Demographic and clinical characteristics of the study group (*N* = 188).

Domain	Trait	Outcome measure	Results
Demographic characteristics	Age	Age (years) Mean (SD)	52.14 (9.83)
	Marital status	Single/married/divorced (%)	Single (10.2)
			Married (67.9)
			Divorced (3.6)
			Widow (14.3)
	Family members	Number of family members Mean (SD)	4.83 (2.23)
	Education	Highest level of Educational level (%)	Primary school (14.8)
			Intermediate and high school (54.1)
			Diploma (16.3)
			University degree (10.7)
	Work	Do you have work? (%)	No (80.6)
			Yes (15.3)
	Monthly income	Level of monthly income (%)	Low income (42.3)			Medium income (51.0)			High income (2.1)
	Disease characteristics	Disease duration	First diagnosis with breast cancer (%)	less than 6 months (14.8)
				6–12 months (18.9)
			more than 12 months (62.2)
Cancer stage		Cancer Stage (%)	(6.6)
		Stage 1	(18.9)
		Stage 2 Stage 3	(40.8)
		Stage 4	(29.6)
Surgery		Surgeries (for breast cancer) (%)	No surgery (12.8)			Have surgery (83.2)

The mean QLI-c total score was (20.22 ± 4.33), indicating overall good QoL score within the sample. The PSQI mean score was (8.16 ± 4.62) indicating poor sleep quality. The mean score of FSFI was (10.9 ± 10.01) indicating a high level of sexual distress. MFIS mean score was (39.92 ± 17.74) indicating a high level of fatigue. The mean score of the HADS-Depression subscale was (7.88 ± 4.4) indicating mild depression. The mean score of the HADS-Anxiety subscale was (7.7 ± 4.2), indicating a borderline of anxiety. The mean of the main outcome measures in this study is shown in Table 2.

**Table 2:** Outcome measures of the study group (*n* = 188).

Trait	Outcome measure	Mean (SD)
Quality of Life	QLI-c	20.22 (4.33)
Depression	HADS depression	7.88 (4.4)
Anxiety	HADS anxiety	7.7 (4.2)
Fatigue	MFIS (unit)	39.92 (17.74)
Sleep Quality	PSQI total score (unit)	8.16 (4.62)
Sexual functioning in women	FSFI	10.9 (10.01)
Physical activity (total)	IPAQ	4634.18 (5671.75)

QLI-c, Quality of Life Index-Cancer; HADS, Hospital Anxiety and Depression Scale; MFIS, Modified Fatigue Impact Scale; PSQI, Pittsburgh Sleep Quality Index; FSFI, Female Sexual Function Index; IPAQ, International Physical Activity Questionnaire.

### 3.2 Correlation results

The findings suggest that monthly income was positively and significantly correlated with QoL (*r* = 0.17, *p* = 0.016). Regarding disease characteristics, disease duration (*r* = −0.280, *p* = 0.004) and BC stage (*r* = −0.221, *p* = 0.002) were negatively correlated with QoL. Regarding impairments, sleep quality (*r* = 0.517, *p* ≤ 0.000), sexual functioning in women (*r* = −0.493, *p* ≤ 0.000), fatigue (*r* = −0.501, *p* ≤ 0.000), depression (*r* = −0.632, *p* ≤ 0.000), and anxiety (*r* = −0.554, *p* ≤ 0.000) were negatively correlated with QoL. Age, marital status, educational level, work status, number of family members, and total physical activity level were not significantly (*p*-value >0.05) associated with the total score of QoL. Table 3 represents Pearson’s correlation.

**Table 3:** Pearson correlations between QOL-C and other variables.

Domain	Trait	QOL-C total score (unit) (All participants (*n* = 188))	
		r	*p*-value
Personal and environmental factors	Age	−0.024	0.742
	Marital status	0.019	0.79
	Education	−0.043	0.558
	Work	0.12	0.089
	Monthly income	0.17	0.016
	Number of family members	−0.123	0.09
Impairments: Disease characteristics	Disease duration	−0.280.	0.004
	Cancer stage	−0.221	0.002
	Surgery	−0.031	0.67
Impairments: Complications	Depression (HADS-Depression)	−0.632^**^	0.000
	Anxiety (HADS-Anxiety)	−0.554^**^	0.000
	Fatigue (MFIS)	−0.501^**^	0.000
	Sleep quality (PSQI)	−0.517^**^	0.000
	Sexual functioning in women (FSFI)	−0.493^**^	0.000
Activity	Physical Activity (IPAQ)	0.123	0.095

HADS, Hospital Anxiety and Depression Scale; MFIS, Modified Fatigue Impact Scale; PSQI, Pittsburgh Sleep Quality Index; FSFI, Female Sexual Function Index; IPAQ, International Physical Activity Questionnaire.

### 3.3 Multivariate regression analysis

Multiple linear regressions were performed to investigate the ability of personal, environmental, and impairment factors to predict QoL in women with BC. In the first model of multiple linear regression, the stage of disease was a predictor of QoL (*t* = −2.003, *p* ≤ 0.047), Table 4.

**Table 4:** Regression analysis of impairments (clinical variables) of QoL-C (*N* = 188).

Trait	*T* value	*p*-value
Cancer stage	−2.003	0.047
Disease duration	−1.689	0.093

The second model of multiple linear regression showed that depression (*t* = −4.339, *p* ≤ 0.000), anxiety (*t* = −3.180, *p* = 0.002), sleep quality (*t* = −2.058, *p* = 0.041), and sexual function (*t* = 2.223, *p* = 0.27) predicting QOL among women with BC (Table 5).

**Table 5:** Regression analysis of Impairment variables (complications) of QoL-C.

Trait	*T* value	*p*-value
HADS-Anxiety	−3.180	0.002
HADS-Depression	−4.339	<0.000
PSQI	−2.058	0.041
MFIS	−1.284	0.201
FSFI	2.223	0.027

HADS, Hospital Anxiety and Depression Scale; MFIS, Modified Fatigue Impact Scale; PSQI, Pittsburgh Sleep Quality Index; FSFI, Female Sexual Function Index; IPAQ, International Physical Activity Questionnaire.

*N* = 188.

The final multiple linear regression model adjusted with age showed that anxiety (*t* = −3.114, *p* = 0.002), depression (*t* = −5.108, *p* < 0.000), sleep quality (*t* = −2.344, *p* = 0.020), and sexual function (*t* = 3.195, *p* = 0.002) were significantly predicting QoL (*p* ≤ 0.05), Table 6.

**Table 6:** Final adjusted regression model.

Trait	*T* value	*p*-value
HADS-Anxiety	−3.114	0.002
HADS-Depression	−5.108	0.000
PSQI	−2.344	0.020
FSFI	3.195	0.002
Age	1.584	0.115

HADS, Hospital Anxiety and Depression Scale; PSQI, Pittsburgh Sleep Quality Index; FSFI, Female Sexual Function Index.

## 4 Discussion

In women, breast cancer is the most prevalent type of cancer. Patients bear a heavy burden after receiving a breast cancer diagnosis, which frequently necessitates making numerous healthcare choices (Shen et al., 2019). This study demonstrated the relationship of ICF components (impairment, activity, participation, and personal factors) to QoL in women with breast cancer. Among these components, the results of this study revealed that the impairment variables of anxiety, depression, sleep quality, and sexual function were found to be the predictors of QoL.

Few studies with conflicting results have concentrated on understanding the factors that affect the quality of life among women with breast cancer (Al-Ghabeesh et al., 2019). In this study we expanded on these previous studies and used the ICF model to examine the intricate interactions between the domains of individual factors, bodily structures and functions, activity, and participation, environmental factors, and health conditions to understand these factors (Shen et al., 2019). This is very important as according to the ICF’s point of view, disability is the result of a conflict between one or more bodily functions and body structures as they are carried out during an activity and the individual’s participation (Anna et al., 2013; Lourenço et al., 2021).

Women with early-stage of breast cancer are twice as likely to experience depression than the general female population, especially in the first year after diagnosis (Anna et al., 2013). An expanded understanding of the psychological-clinical implications is made possible by the addition of the ICF evaluation to the standard psychological assessment (Anna et al., 2013). Our study showed that depression and anxiety predict QoL. These results support the results of previous studies. For example, a study of 90 women with BC in Korea showed that depression was negatively associated with QoL (Han et al., 2019). Another study done by Hsiao, F.-H., et al. found that high anxiety symptoms and increased depression symptoms were associated with overall QoL deterioration in Chinese BC women (Hsiao et al., 2019). Additionally, depression was a significant predictor of general and specific QoL. Furthermore, breast cancer patients experiencing menopausal symptoms and reduced emotional well-being faced an increased likelihood of experiencing anxiety and depression. (Han et al., 2019; Shim et al., 2020). These findings confirmed the need for psychosocial support programs to improve psychological well-being in BC women which might improve the QoL. These findings underscore the necessity for psychosocial support programs tailored to improve the psychological well-being of women with breast cancer. These initiatives might encompass a range of supportive measures, including counseling, therapy sessions, support groups, and educational programs aimed at addressing the emotional and psychological challenges faced by women dealing with breast cancer (Hodge and Lonsdale, 2011; Turner et al., 2011). Including exercise within these programs could significantly alleviate emotional distress and boost overall QoL in this population (Burke et al., 2012). Exercise not only tackles psychological challenges but also enhances physical well-being, positively impacting mood, reducing stress, and improving mental health (Burke and Sabiston, 2010; Durosini et al., 2021).

According to our findings, poor sleep quality was negatively associated with QoL, which supports the findings of previous studies (Liu et al., 2013; Sanford et al., 2013; Al Maqbali et al., 2022). In a cross-sectional study involving 133 women diagnosed with breast cancer, researchers found a correlation between heightened sleep disturbances and a decline in QoL (Al Maqbali et al., 2022). A study by Liu et al. found that poor sleep quality that was measured subjectively and objectively was significantly associated with poor QoL in BC patients who received adjuvant chemotherapy (Liu et al., 2013). Stanford et al. also confirmed that sleep quality exhibited a consistent negative correlation with QoL throughout all stages of chemotherapy (Sanford et al., 2013). In addition, a study in Taiwan reported that in addition to pain, stress, and psychological deficits sleep disturbances might predict QoL in women with BC (Weng et al., 2021). In our study sample, the diminished sleep quality could be linked to post-adjuvant treatment symptoms like pain, fatigue, and hot flashes, potentially leading to decreased QoL in these women. Hence, it’s essential for healthcare providers treating individuals with breast cancer to take into account their sleep quality. Assessing sleep in this population is very important. Furthermore, implementing interventions to improve sleep is deemed critical. Research in Jordan has primarily focused on group educational lectures, which have shown effectiveness in enhancing breast health knowledge and overall QoL (Taha et al., 2010; Alsaraireh and Darawad, 2019). However, it’s important to note that no studies have explored the impact of any interventions on sleep quality and, consequently, on QoL. Cognitive Behavioral Therapy (Ma et al., 2021), aerobic training (Courneya et al., 2013), musical therapy (Chang et al., 2021), and resistance training (Dieli-Conwright et al., 2021) were found to be effective interventions to improve sleep quality in women with BC. Subsequent research efforts are needed to implement these interventions within the context of Jordanian culture, as no prior studies have explored this specific type of intervention in Jordan.

Approximately 20% of breast cancer patients experience sexual impairment, a statistic corroborated by numerous studies indicating the challenges faced by these patients in engaging in sexual activity (Jamshidi et al., 2022). To our knowledge, this is the first study to examine the effect of sexual function on QoL among Jordanian women with BC. Using ICF model, the finding of this current study also showed that sexual function was a significant predictor of QoL. Our results are in line with previous studies (Park and Yoon, 2013) reported a significant link between the sexual function of BC women and QoL. Safarinejad et al. reported similar findings in their study on 390 women with BC (Safarinejad et al., 2013). A potential explanation of these findings could be the interaction between BC treatment, lack of sexual desire and attractiveness, and worse relationships with the partner. A previous study found that the deterioration of sexual QoL in patients with BC was associated with increased anxiety and depression levels, body image disturbed, and lower self-esteem in these patients (Brajkovic et al., 2021). Furthermore, the poor social support of partners had a negative effect on sexual QoL in breast cancer women (Brajkovic et al., 2021). Therefore, these findings suggest that targeted interventions to improve sexual function may help sexual health recovery and improve QoL in women with BC.

In fact, the ICF has affirmed the significance of taking a broad view of a person’s life, identifying, describing, and categorizing a person’s health condition with respect to the individual and society as a whole rather than just with respect to the functional profile or the subjective perception of well-being (Anna et al., 2013). This study highlights the importance of factors considered long-term challenges faced by Jordanian women with BC and their influence on QoL in those women. Understanding the relationship between these impairments (such as level of sleep quality, sexual function status, and current status of anxiety and depression) and QoL will allow healthcare professionals to offer individualized intervention to those women who had a high probability of poor QoL. These findings support the need for more longitudinal studies of demographic, behavioral, social, and clinical factors that correlate with QoL for different breast cancer populations in a larger sample size. The ICF is still a vital tool for assessing how well BC survivors are doing in their rehabilitation (Pinto et al., 2022).

It must be acknowledged that our study has some limitations. The study was a cross-sectional design that is considered observational, leaving the issue of causality ambiguous. Furthermore, using a convenience method of the population sample makes complete generalization not possible when using this design. In this study, we lacked sufficient information regarding the specific surgery performed on the patients. We hypothesize that various types of breast surgeries may result in distinct impacts on social health. Further research is necessary to document this information and investigate its effects. Additionally, most of participating women were not workers and had intermediate school education. Therefore, the study participants may not be representing working women and women with high education.

In summary, this study investigated the factors that might impact the QoL of Jordanian women with breast cancer. The utilization of the ICF model help to understand these factors. Among all ICF components, the results of this study revealed that the impairment variable (anxiety, depression, sleep quality, and sexual function) was the predictor of QoL. This understanding enables the development of personalized care plans that address specific challenges faced by patients, potentially leading to more effective treatment outcomes and a better overall experience throughout their cancer journey.

## Data availability statement

The datasets presented in this article are not readily available because the data that support the findings of this study are available upon request from the corresponding author. The data cannot be publicly available due to privacy and ethical considerations. Requests to access the datasets should be directed to AA-Sh, aal-sharman@sharjah.ac.ae.

## Ethics statement

The studies involving humans were approved by Institutional Review Board (IRB) at the Jordan University of Science and Technology (JUST), IRB at the King Abdullah University Hospital (KAUH), and the Scientific Research Ethics Committee at the Jordanian Ministry of Health. The studies were conducted in accordance with the local legislation and institutional requirements. The participants provided their written informed consent to participate in this study.

## Author contributions

AA-Sh: Project administration, Validation, Visualization, Writing – original draft, Writing – review & editing, Data curation, Formal analysis, Conceptualization, Funding acquisition, Investigation, Methodology, Resources, Software, Supervision. AA-Sa: Investigation, Resources, Writing – original draft, Data curation, Formal analysis, Methodology, Software. AA: Data curation, Formal analysis, Investigation, Methodology, Resources, Software, Writing – original draft. RS: Data curation, Formal analysis, Investigation, Methodology, Resources, Software, Writing – original draft. AB-A: Data curation, Formal analysis, Writing – original draft, Investigation, Methodology, Resources, Software. CS: Data curation, Formal analysis, Investigation, Methodology, Resources, Software, Writing – original draft. WB: Writing – review & editing. DA: Formal analysis, Visualization, Writing – review & editing. HH: Writing – review & editing. HK: Conceptualization, Data curation, Formal analysis, Investigation, Methodology, Project administration, Resources, Software, Supervision, Validation, Visualization, Writing – original draft.
